# The Hygienic Relations of Artificial Dentures

**Published:** 1883-04

**Authors:** 


### ARTICLE II.

### *The Hygienic Relations of Artificial Dentures.*

Editor of The American Journal of Dental Science.

*Dear Sir:*—Allow me to make a few remarks on Dr. Kirk's article, in Dental Cosmos, for March 1883, on the above subject, in the hope that the readers of your valuable Journal may contribute their experience in the matter and give it the publicity so much needed.

After twenty-two years experience I have found that there is no best material for artificial dentures, while some can wear vulcanite, others will have their mouths ulcerated after wearing a plate of it for a week. In metal anything under 18 carat is unreliable, 16 carat gold turning quite black, and in the same mouth a vulcanite plate has been worn with comfort and without injurious effect for ten years. I have seen some of the rubber pieces after two years wear, turn so soft that they had the appearance of leather, other rubber bases had absorbed the secretions so thoroughly that not even strong acids would remove the foul odor that they emitted.

I may mention that it depends entirely upon the preparation of vulcanite whether it is fit for the mouth or not. I have a vulcanite piece in my mouth made in the year 1870, and it is at this moment as free from odor as the day it was made; while I have never experienced the slightest inconvenience, and the tissues have remained healthy throughout this long period. It is made of pure black rubber, (not colored.) faced with Ash's 11 pink, and I attribute its durability to the care in working it. It was vulcanized first for a long time, say one hour and a half in plenty of steam at 250° Far. and finished off at 300° for two hours. Its surface is very little changed and has never been repolished; my only treatment is washing daily in warm soap and water with the addition of a little chalk. A most important point in vulcanizing is, always use a clean boiler for each steaming.

I have found that over cooked rubber is very injurious, becoming very absorbent and causing a constant irritation. Another cause of rubber being condemned is, it is often put in the mouth in a rough unfinished state, causing great irritation, which sometimes has had the appearance of saturation, while the same piece being properly trained and highly polished, has given no further trouble. Of all metals that I have found giving the best result, platina with pure gold solder stands first. By careful manipulation it can be made to hold by suction independent of its extra weight, and highly polished plates of this material, I have seen, after fifteen years, giving the greatest satisfaction, both to myself and patient.

Continuous gum work for complete sets, also gives the best results, after six years wear the mouth remains healthy and very little absorption is noticable.

Celluloid is not thoroughly reliable unless made between metal moulds and under a heavy pressure; if pressed between plaster moulds it is impossible to get the surface sufficiently hard; no amount of polish will resist the action of the fluids of the mouth, unless celluloid is protected by that coat or surface that steaming between metal dies gives to it. It is the material that absorbs the fluids of the mouth quicker than any other dental plate, while for a smoker it is useless, turning in a short time completely black or brown throughout the denture and emitting an offensive odor; while vulcanite will be found only coated or glazed and can be made clean again by scraping and polishing.

*Celluloid Cannot be Restored.*

A combination such as gold and vulcanite, dental alloy with silver solder, and low standard gold I have noticed always cause an inflamed state of the mouth. The first by the two materials not uniting, allowing a deposit to accumulate between the plate and rubber base and becoming decomposed, the latter by their being quickly acted upon by weak acids causing a metallic taste and fermentation of certain foods deposited upon them during the day, and the rapid decomposition of the inferior metals used as solder for low standard metals.

G. A. D. G.

